# Comparison of influenza disease burden in older populations of Hong Kong and Brisbane: the impact of influenza and pneumococcal vaccination

**DOI:** 10.1186/s12879-019-3735-7

**Published:** 2019-02-14

**Authors:** Lin Yang, King Pan Chan, Chit Ming Wong, Susan Shui Seng Chiu, Ricardo J. Soares Magalhaes, Thuan Quoc Thach, Joseph Syrial Malik Peiris, Archie C. A. Clements, Wenbiao Hu

**Affiliations:** 1 GH515, School of Nursing, The Hong Kong Polytechnic University, Hung Hom, Hong Kong, Hong Kong, Special Administrative Region of China; 2 School of Public Health and Social Work, Queensland University of Technology, Brisbane, Australia; 3 School of Public Health, The University of Hong Kong, Hong Kong, Hong Kong, Special Administrative Region of China; 4 Department of Pediatrics and Adolescent Medicine, The University of Hong Kong, Hong Kong, Hong Kong, Special Administrative Region of China; 5 School of Veterinary Science, University of Queensland, Brisbane, Australia; 6 Research School of Population Health, The Australian National University, Canberra, Australia

**Keywords:** Influenza, Disease burden, Mortality, Morbidity, Elderly, Vaccine

## Abstract

**Background:**

Influenza and pneumococcal vaccine uptake in the older population aged 65 years or over of Hong Kong dramatically increased since the 2003 SARS outbreak. This study is aimed to evaluate the impact of increased coverage of influenza and pneumococcal vaccines by comparing the change of disease burden in the older population of Hong Kong, with the burden in the older population of Brisbane with relatively high vaccine coverage in the past fifteen years.

**Methods:**

Time series segmented regression models were applied to weekly numbers of cause-specific mortality or hospitalization of Hong Kong and Brisbane. Annual excess rates of mortality or hospitalization associated with influenza in the older population were estimated for the pre-SARS (reference period), post-SARS and post-pandemic period, respectively. The rate ratios (RRs) between these periods were also calculated to assess the relative change of disease burden.

**Results:**

Compared to the pre-SARS period, excess rates of mortality associated with influenza during the post-SARS period in Hong Kong decreased for cardiorespiratory diseases (RR = 0.90, 95% CI 0.80, 1.01), stroke (RR = 0.74, 95% CI 0.50, 1.09), and ischemic heart diseases (RR = 0.45, 95% CI 0.34, 0.58). The corresponding RRs in Brisbane were 0.79 (95% CI 0.54, 1.15), 0.33 (0.13, 0.80), and 1.09 (0.62, 1.90), respectively. Only the mortality of ischemic heart diseases showed a greater reduction in Hong Kong than in Brisbane. During the post-pandemic period, excess rates of all-cause mortality increased in Hong Kong, but to a lesser extent than in Brisbane (RR = 1.41 vs 2.39).

**Conclusion:**

A relative decrease (or less of an increase) of influenza disease burden was observed in the older population of Hong Kong after increased coverage of influenza and pneumococcal vaccines in this population, as compared to those of Brisbane where vaccination rates remained stable. The lack of significant findings in some disease categories highlights the challenges of evaluating the benefits of vaccination at the population level.

**Electronic supplementary material:**

The online version of this article (10.1186/s12879-019-3735-7) contains supplementary material, which is available to authorized users.

## Background

Globally, influenza has been associated with a heavy burden of mortality and morbidity [1]. Vaccination remains an important strategy to reduce disease severity and virus transmission within the community [2]. Although numerous clinical trials have demonstrated the effectiveness of influenza vaccines in children [3], adults [4], and healthy elderly people [5], few studies have included high-risk groups particularly the elderly with underlying chronic conditions. A recent systematic review also concluded that influenza vaccine only had a modest effect in preventing influenza infections among community-dwelling elderly people [6]. Previous cohort or case-control studies reported that vaccine effectiveness was 48% in preventing all-cause mortality [7]. However, according to a study in the US, < 5% of all-cause mortality was specifically associated with influenza, suggesting that the estimates from the observational studies could have been seriously overestimated [8, 9]. Another ecological study conducted in Ontario, Canada also found significant relative reductions in influenza-associated mortality and health care utilization after the introduction of universal vaccination since 2000 in those aged < 65 yrs., but not in those aged ≥65 yrs. [10]. Taken together, available evidence suggests that there is a need to assess the effect of influenza vaccination at the population level, especially for those aged ≥65 years.

Previous studies in Hong Kong have shown that annual vaccination rates for community-dwelling elderly people were less than 3% during the period 2000–2002 [11], but increased to more than 50% in 2004–2006 [12]. Since October 2009, a subsidy of HK$80 (US$10.30) for annual influenza vaccine and HK$190 (US$24.50) for pneumococcal vaccine has been provided to those aged ≥65 years under the Elderly Vaccination Subsidy Scheme. The vaccination rate remained nearly 40% in the elderly in the 2012/13 season [13]. In Australia, the federal government has been providing free influenza vaccinations for people aged ≥65 years since 1999, and the coverage rates in the older population remained between 70 and 80% during the period of 2002–2006 [14]. Unlike Hong Kong, where the SARS outbreak and a new subsidy program greatly increased influenza and pneumococcal vaccine coverage among the older population, Brisbane has had a relatively stable vaccination rate for both vaccines since 2000. Here we hypothesize that if influenza vaccine was effective in older people, the dramatically increased uptake among the older population of Hong Kong since SARS could have resulted in a reduced influenza disease burden. We expect such a reduction to be larger than in Brisbane, where uptake of the vaccine has remained stable among the community dwelling elderly people. Further decrease in disease burden of influenza could have occurred after 2009, as the increased uptake of pneumococcal vaccines in the older population could have reduced the risk of secondary bacterial pneumonia after influenza infections.

## Methods

### Study population

Hong Kong is located at a latitude of 22°N, with a population of 6.9 million in 2006 residing in an area of 1104 km^2^. Brisbane is located at a latitude of 27°S, with a population of 1.8 million in 2006 and a territory of 1360 km^2^. Both Hong Kong and Brisbane have a subtropical climate, with average temperatures of 27 °C and 20 °C, and relative humidity of 80 and 60%, respectively. In terms of socioeconomic conditions, both are developed cities with a comparable Gross Domestic Product per capita ($31,514 in Hong Kong vs. $55,671 in Australia in 2010) [15]. During the study period, the percentage of the population aged ≥65 years was 12.5% in Hong Kong and 11.6% in Brisbane. Virology data, death registry data, hospital admission, and meteorological data during the study period of 2001 to 2012 were obtained from difference data sources of Hong Kong and Brisbane, respectively. The detailed information is provided in Additional file 1: Appendix 1.

#### Study period

**Fig. 1:**
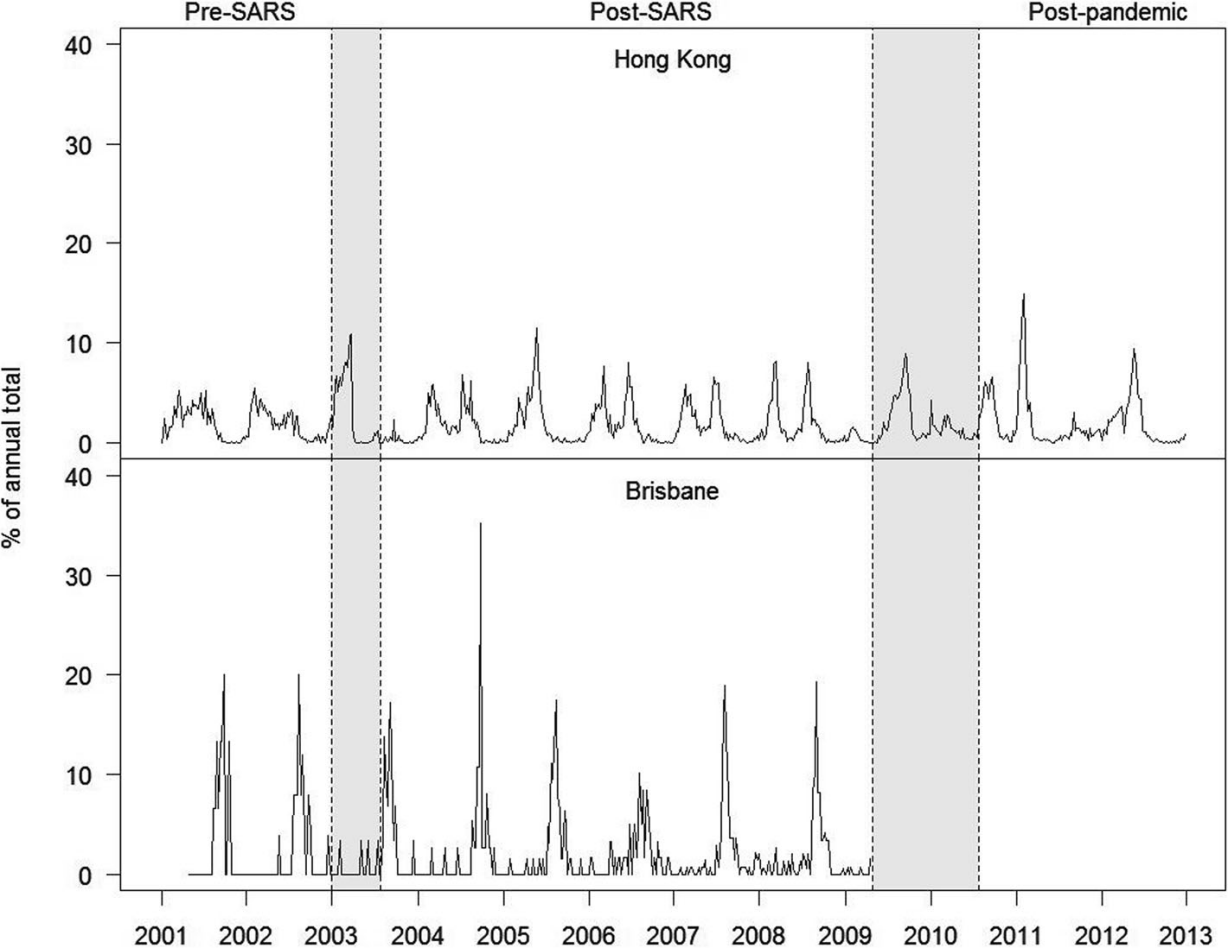
Time series plots of laboratory confirmed influenza in Hong Kong (upper panel) and Brisbane (lower panel), 2001–2012. The gray bands highlight the SARS period and the 2009 H1N1 pandemic

In Hong Kong, seasonal influenza peaks during January – March and June – July, whereas in Australia the peak usually occurs in August – October (Fig. 1). Given that seasonal influenza peaks at different times in these two cites, we defined annual study period as January – December in Hong Kong and May – April of next year in Brisbane. These periods begin three months after the usual launch dates for the annual seasonal influenza vaccination campaigns (March in Brisbane and September in Hong Kong), which shall allow for a valid assessment of the vaccination effectiveness. The whole study period was divided into the pre-SARS, SARS, post-SARS, influenza pandemic and post-pandemic periods. The pre-SARS period in Hong Kong was featured with a much lower vaccination rate in the older population compared to the post-SARS and post-pandemic periods, whereas the vaccination rate in the older population of Brisbane was stable across these periods. The burden during the SARS and pandemic periods was not presented, as this was highly affected by different control measures adopted by the health authorities of Hong Kong and Brisbane. The cut-off dates for these periods in Hong Kong and Brisbane are listed in Additional file 1: Appendix 2.

#### Statistical analysis

We constructed time series segmented regression models to estimate cause-specific mortality or hospitalization risks associated with influenza in the older population during the pre-SARS, post-SARS, and post-pandemic periods for Hong Kong and Brisbane. The proxy variable for influenza in the model was the percentage of specimens that tested positive for influenza each week out of annual total number in each city. The reason why we decided to use annual percentage instead of weekly proportion used in our previous studies is that total numbers of specimens were not available in Brisbane during the whole study period. We added seasonal trends, temperature, humidity, and other respiratory viruses to the model as covariates to estimate influenza-associated excess risks. Dummy variables for the pre-SARS, post-SARS, and post-pandemic periods, together with the interaction terms between these period dummies and the virus activity variables, were also added to test the statistical differences in risk estimates between the different periods, respectively for Hong Kong and Brisbane.

The best-fit models were chosen by the minimal generalized cross-validation (GCV), according to our previous study [16]. Baseline rates of cause-specific mortality and hospitalizations associated with influenza were calculated for different periods by setting the virus proxy to zero and the corresponding period dummy to one (other dummies were simultaneously set to zero). We first estimated excess numbers by subtracting baseline rates from the observed data, and calculated excess rates (ER) by dividing the excess numbers with age-specific population size.

We calculated the 95% confidence interval (CI) of ER by bootstrapping 1000 times. Because the periods were of different lengths, annual excess rates (AER) of mortality (or hospitalizations) were calculated to facilitate comparisons between different periods. For each disease category, the rate ratios (RRs) of post-SARS (or post-pandemic) versus pre-SARS were derived by dividing annual excess rates during the post-SARS (or post-pandemic) period with those of the pre-SARS period (as reference):

*RR = AER (post-SARS) / AER (pre-SARS).*

Since the pre-SARS period was treated as the reference period in this study, hereafter the post-SARS RR refers to the risk ratio of mortality or hospitalization in the post-SARS period relative to those in the pre-SARS period. Similarly, the post-pandemic RR refers to the risk ratio of mortality or hospitalization in the post-pandemic period relative to those in the pre-SARS period. The 95% CI and *p*-value of RR were derived from a normal approximation of their logarithmic transformations [17].

We also conducted a subset analysis by using the data of influenza peak seasons only. The influenza season was defined as January to July in Hong Kong, and May to November in Brisbane. We conducted another subset analysis by excluding the data for the mismatched years (2003, 2004, and 2008 in this study).

All of the analyses were conducted in R software version 2.5.1. The significance level was set to 0.05 for all analyses.

## Results

During the study period, there were around 860,000 and 112,000 people older than 65 years living in Hong Kong and Brisbane, respectively (Tables 1 and 2). Compared to Hong Kong, during the study period Brisbane had higher mortality rates for all-cause (81.7 vs 66.5 per 100,000 population), cardiorespiratory diseases (CRD, 42.1 vs 33.8), stroke (9.5 vs 6.5) and ischemic heart diseases (IHD, 17.0 vs 7.5), but a lower rate for pneumonia and influenza (P&I, 2.8 vs 9.9), and a comparable rate for chronic obstructive pulmonary disease (COPD, 3.9 vs 4.2) (Additional file 1: Appendix 3). All the cause-specific hospitalization rates were much lower in Brisbane than in Hong Kong (233.6 vs 411.7 per 100,000 population for CRD, 21.7 vs 69.5 for P&I, 39.7 vs 79.9 for COPD, 19.4 vs 73.8 for stroke), with the only exception of IHD (65.1 vs 76.6) (Additional file 1: Appendix 4). Hong Kong and Brisbane have opposite seasonal climate patterns; the former is located in the Northern Hemisphere and the latter in the Southern Hemisphere. Mean temperature and relative humidity of Brisbane were lower than those of Hong Kong (Additional file 1: Appendix 5).

**Table 1:** Summary statistics of demographic characteristics, mortality, hospitalization, and virological and meteorological data in Hong Kong in different periods

	Whole period				Pre-SARS				Post-SARS				Post-pandemic			
	Mean	SD	Min.	Max	Mean	SD	Min.	Max	Mean	SD	Min.	Max	Mean	SD	Min.	Max
Elderly population (per 1000)	860.4	65.5	753.3	980.3	765.2	12	753.3	777.1	850.6	28.8	795.4	898.6	953.5	24	918.5	980.3
Weekly death no. (n)																
All-causes	572.5	86.6	406	869	488.6	49.5	406	624	569.5	76.3	415	869	630.8	85.3	484	869
CRD	290.7	58.8	179	522	240.5	35.8	179	351	291.4	54.5	194	522	321.3	60.9	234	503
P&I	85.3	28	36	190	55.1	9.6	36	98	80.8	21	41	186	115.9	25.4	71	190
COPD	36	9.6	12	67	37	9.3	19	64	36.2	9.7	12	65	34.2	9.4	19	67
Stroke	55.6	10.9	30	92	52.8	10.6	32	81	57.4	10.8	30	92	52.8	10.6	30	84
IHD	64.1	15.4	34	129	53	10.8	34	82	66.2	15.1	37	129	66.4	15.7	35	120
Accidents	10.7	3.7	2	23	10.2	3.7	2	20	10.8	3.8	3	23	10.2	3.4	4	20
Weekly hospitalization no. (n)																
CRD	3542.5	958.5	1704	6340	3196.9	283.2	2535	3945	2965.3	445.4	2260	4566	4858.7	485.1	3906	6079
P&I	597.9	306.7	98	1497	177	34	111	256	592.7	187.8	124	1200	930.5	199.6	673	1497
COPD	687.8	168.6	285	1188	767.3	104.4	576	1040	596.2	112.4	387	1048	815.9	147.6	573	1188
Stroke	635.3	211.6	329	1254	611.5	60.1	471	750	479.9	79.8	334	766	917.7	92.5	723	1166
IHD	659.4	225.1	257	1368	583.7	63	471	718	516.3	90.1	374	866	958.4	109.7	711	1279
Accidents	493.2	115.4	267	845	430.6	39.3	354	547	434	61.8	325	634	656.8	55.1	549	845
Weekly positive specimens (n)	19.8	32.8	0	310	11.8	9.7	0	41	13.6	15.8	0	93	23.9	30.9	0	157
Weekly positive percentage (%)	1.9	2.2	0	14.9	1.9	1.5	0	5.6	1.7	2	0	11.5	2.1	2.7	0	14.9
Temperature (°C)	23.5	4.9	11.4	30.5	23.8	4.5	14.1	30.2	23.2	4.8	11.4	30.5	23.4	5.2	12.3	30.1
Relative Humidity (%)	78.3	7.8	51	93.7	78.5	7.5	54.4	92	77.7	8.3	51	93.6	77.9	7.5	54.9	93.7

Note. Weekly positive percentage (%) is defined as the percentage of positive specimens among annual total specimens

**Table 2:** Summary statistics of demographic characteristics, mortality, hospitalization, and virological and meteorological data in Brisbane in different periods

	Whole period				Pre-SARS				Post-SARS				Post-pandemic			
	Mean	SD	Min.	Max.	Mean	SD	Min.	Max.	Mean	SD	Min.	Max.	Mean	SD	Min.	Max.
Elderly population (per 1000)	116.2	6.5	106.6	129	107.8	1	106.6	108.6	114	2.6	110.4	119.8	126.1	2.5	122.7	129
Weekly death no. (n)																
All-causes	94.9	13.7	56	142	94.6	13.9	74	134	95	13.6	63	142	95	15.5	56	141
CRD	48.9	10.3	23	85	53.6	11.4	31	85	50	10.1	23	80	43.6	9.3	26	76
P&I	3.2	2	0	13	3.6	1.9	0	11	3.8	2.1	0	13	2.3	1.6	0	8
COPD	4.5	2.3	0	15	4.9	2.5	0	11	4.3	2.3	0	15	4.7	2.3	0	12
Stroke	11	3.7	2	29	13.6	4	5	29	11.1	3.6	3	22	9	2.9	2	18
IHD	19.7	5.8	6	45	22.4	6.3	9	45	20.2	5.6	6	38	16.6	4.8	7	35
Weekly hospitalization no. (n)																
CRD	271.4	45.7	189	467	266.4	44.9	193	384	275.3	46.3	189	467				
P&I	25.2	10.6	9	65	24.7	10	9	51	25.9	10.8	9	65				
COPD	46.1	14.8	18	96	45.3	14.8	18	87	47.2	15.1	20	96				
Stroke	22.5	5.1	9	37	22.5	4.8	10	36	22.6	5.2	9	37				
IHD	75.7	10.3	43	103	78.6	9.8	57	103	74.7	10.6	43	102				
Weekly positive specimens (n)	6.1	22.1	0	272	0.5	0.9	0	5	1.6	3.5	0	28	23.8	44.3	1	272
Weekly positive percentage (%)	2	3.6	0	35.1	2.3	4.6	0	20	2	3.9	0	35.1	2.1	2.4	0	13.2
Temperature (°C)	21.2	3.7	13.4	30.4	20.8	3.8	14.3	28.4	21.6	3.7	13.5	30.4	20.9	3.5	14.1	27.8
Relative Humidity (%)	57.9	8.5	32.1	90.2	54.4	7.7	36.1	73.9	57.4	8.1	32.1	79.2	60.4	9.2	36.9	90.2

Note. Weekly positive percentage (%) is defined as the percentage of positive specimens among annual total specimens

Compared to the pre-SARS period, average number of hospitalizations in Hong Kong was lower in the post-SARS period for most disease categories, with the exception of a three-fold increase in P&I hospitalizations. This significant increase is likely due to the change in coding practice after the SARS outbreak (Table 1). Average number of hospitalizations was higher in the post-pandemic period for all the disease categories. Weekly figures for mortality and hospitalization in Brisbane did not obviously differ across the pre-SARS, post-SARS, and post-pandemic periods (Table 2).

Due to negative estimates in the annual excess rates for mortality, the post-SARS RR could not be estimated for IHD hospitalizations in Hong Kong, and P&I mortality, stroke, and IHD hospitalizations in Brisbane. Similarly, the post-pandemic RR could not be estimated for P&I and IHD hospitalization in Hong Kong, and stroke and COPD hospitalization in Brisbane (Additional file 1: Appendix 6). Influenza-associated all-cause mortality rates were found increased after SARS in Hong Kong (post-SARS vs pre-SARS RR = 1.22) but decreased in Brisbane (RR = 0.87). The post-SARS COPD mortality increased in both cities (1.04 and 1.03 in Hong Kong and Brisbane, Table 3). Decreased excess rates of mortality in Hong Kong were observed for CRD, stroke, and IHD mortality (RR = 0.90 and 0.74), while the corresponding RRs in Brisbane were 0.79 and 0.33, respectively. Only IHD mortality had a greater reduction observed in Hong Kong than in Brisbane (RR = 0.45 vs 1.09). None of RR estimates for the control disease injuries were significant (data not shown).

**Table 3:** Rate ratio (RR) of excess mortality or hospitalization associated with influenza between the post-SARS and pre-SARS (reference) periods in Hong Kong and Brisbane; and the ratio of RR (RRR) between Hong Kong (HK) and Brisbane

	Whole period				Peak seasons only^aPeak seasons onlya^				Vaccine matched years onlybVaccine ^matched years onlyb^			
	Hong Kong		Brisbane		Hong Kong		Brisbane		Hong Kong		Brisbane	
	RR	95% CI	RR	95% CI	RR	95% CI	RR	95% CI	RR	95% CI	RR	95% CI
Mortality Post-SARS vs Pre-SARS												
All-causes	1.22	(1.10,1.35)	0.87	(0.62,1.21)	1.48	(1.35,1.63)	0.93	(0.72,1.19)	2.62	(2.25,3.05)	4.39	(2.67,7.22)
CRD	0.90	(0.80,1.01)	0.79	(0.54,1.15)	1.11	(1.00,1.23)	3.54	(2.23,5.62)	1.25	(1.08,1.45)	4.75	(2.55,8.83)
P&I	2.50	(2.00,3.13)	NE		1.26	(1.06,1.49)	NE		1.53	(1.23,1.91)	NE	
COPD	1.04	(0.78,1.38)	1.03	(0.44,2.39)	NE		1.71	(0.54,5.37)	NE		1.39	(0.62,3.13)
Stroke	0.74	(0.50,1.09)	0.33	(0.13,0.80)	NE		0.31	(0.14,0.71)	1.18	(0.81,1.71)	0.58	(0.28,1.20)
IHD	0.45	(0.34,0.58)	1.09	(0.62,1.90)	0.48	(0.40,0.58)	1.36	(0.81,2.27)	0.16	(0.11,0.23)	NE	
Hospitalization Post-SARS vs Pre-SARS												
CRD	0.86	(0.82,0.91)	26.82	(9.56,75.24)	5.25	(4.86,5.68)	NE		15.83	(13.50,18.56)	28.09	(9.80,80.53)
P&I	2.79	(2.57,3.03)	3.49	(1.96,6.21)	5.18	(4.74,5.67)	NE		6.37	(5.65,7.19)	2.02	(1.20,3.40)
COPD	1.04	(0.96,1.14)	1.33	(0.91,1.93)	9.12	(7.77,10.71)	1.35	(0.96,1.89)	NE		1.10	(0.76,1.59)
Stroke	0.15	(0.12,0.19)	NE		0.02	(0.01,0.05)	NE		NE		NE	
IHD	NE		NE		NE		NE		NE		NE	
Mortality Post-pandemic vs Pre-SARS												
All-causes	1.41	(1.28,1.56)	2.39	(1.83,3.12)	1.16	(1.06,1.28)	2.06	(1.67,2.55)	2.96	(2.55,3.44)	7.21	(4.48,11.62)
CRD	1.16	(1.04,1.29)	0.98	(0.69,1.38)	1.06	(0.96,1.18)	4.10	(2.61,6.44)	1.48	(1.29,1.71)	2.19	(1.12,4.27)
P&I	2.44	(1.95,3.05)	NE		1.51	(1.28,1.77)	NE		1.69	(1.37,2.09)	NE	
COPD	1.07	(0.81,1.41)	1.75	(0.83,3.67)	NE		7.42	(2.82,19.54)	NE		1.86	(0.87,3.96)
Stroke	1.26	(0.90,1.77)	0.29	(0.12,0.72)	NE		0.77	(0.42,1.39)	1.43	(1.01,2.03)	0.43	(0.20,0.93)
IHD	0.74	(0.59,0.92)	NE		0.34	(0.27,0.41)	NE		0.40	(0.31,0.51)	NE	

Note: NE, not estimated due to negative estimates in annual excess rates.The p-value of the RRR of Hong Kong versus Brisbane was calculated from z-tests

a Influenza peak season is January to July in Hong Kong, May to November in Brisbane

b The data of years 2003, 2004 and 2008 were excluded due to mismatch between vaccine composite and circulating influenza strains

Compared to the pre-SARS period, excess rates of CRD hospitalizations in the post-SARS period decreased in Hong Kong, but increased markedly in Brisbane (RR = 0.86 vs 26.82). Influenza-associated hospitalizations for P&I and COPD increased more in Brisbane than in Hong Kong (RR = 3.49 vs 2.79 and 1.33 vs 1.04).

Compared to the pre-SARS period, excess mortality rates increased in Hong Kong for all the disease categories except for IHD, but only all-cause and COPD mortality increased in Brisbane. Difference between Hong Kong and Brisbane was found statistically significant for all-cause and stroke mortality. Annual excess rates of all-cause mortality increased in Hong Kong to a lesser extent than in Brisbane (RR = 1.41 vs 2.39), whereas an opposite trend was observed for stroke mortality (RR = 1.26 vs 0.29).

Subset analysis with peaks seasons only, or vaccine matched years only, generally derived larger RR estimates (Table 3). The estimates for CRD became significantly higher than one, and the Brisbane estimates were much greater than the Hong Kong ones. Many outcomes could not be estimated due to negative values of excess rates.

## Discussion

In this study, we estimated excess rates of mortality or hospitalizations attributable to influenza in different periods (pre-SARS, post-SARS, and post-pandemic) for two subtropical cities Hong Kong and Brisbane. We hypothesized that the influenza disease burden decreased more, or increased less, in Hong Kong than in Brisbane since 2003, because the uptake rate of influenza and pneumococcal vaccines increased more markedly in Hong Kong than in Brisbane during the same period. Kwong et al. [10] compared the relative change of disease burden in Ontario, where a universal influenza vaccination program was launched, to that in other Canadian provinces without such a policy. They found that influenza-associated mortality fell in Ontario and other provinces, but a larger reduction occurred in Ontario. In this study, we found that excess rates of IHD mortality decreased more from the pre-SARS to the post-SARS period in Hong Kong than in Brisbane, but with regard to the other mortality outcomes, excess rates increased more in Hong Kong. With respect to hospitalization in the post-SARS period, significantly lower excess rates were only found for CRD in Hong Kong. P&I and COPD hospitalization rates increased in both cities, but to a lesser extent in Hong Kong.

Many countries have recommended annual influenza vaccination or providing subsidy programs to the older population [18]. However, due to ethical concerns, a large body of knowledge on the effectiveness of influenza vaccine in the older population has been derived from observational studies, as few randomized controlled trials have been conducted in this high-risk population. A review by Goodwin et al. found that seroprotection and seroconversion achieved in the older population after vaccination was only 25–50% of vaccine response in younger adults [19]. A Cochrane review concluded that influenza vaccines were of limited effects, which could probably be explained by weak antibody response in the older population [6]; however, in a recent reanalysis using the same data, Walter et al. made the opposite conclusion [20]. They estimated that influenza vaccine resulted in a 30% reduction in complications after influenza infections, 40% in influenza-like illnesses, and 50% in laboratory confirmed influenza infections, specifically during influenza epidemics. However, there is still an on-going debate on vaccine effectiveness in the older population. Our findings add some evidence of a decrease, or a slow increase, in influenza-associated disease burden among the older population, following a marked increase in influenza vaccine coverage at the population level. However, it should be noted that this effect could have been partially caused by an increase of pneumococcal vaccination at the same time. Given that the effectiveness of influenza vaccine is affected by many factors including pre-existing immunity, antigenic shift, and underlying condition, it is not surprising that we failed to find consistent and significant estimates. This also highlights the challenges of evaluating the benefits of vaccination at the population level, even in the most susceptible older populations.

A higher disease burden was found in both Hong Kong and Brisbane after 2003, which was consistent with the findings of our previous studies and others [21, 22]. The potential explanation could be that H3N2 was more often predominant after 2003 and this subtype has been found associated with higher disease burden than H1N1 and B. The point estimates of RRs were sensitive to modeling parameters, and most had wide confidence intervals. This could be due to only a relatively small proportion of deaths or hospitalizations attributable to influenza. According to our previous studies, each year influenza is associated with nearly 1000 deaths and 10,000 hospitalizations in Hong Kong, accounting for only 3 and 1% of annual total deaths and hospitalizations, respectively [22–25]. Negative estimates of excess rates were occasionally derived from some disease outcomes, making it difficult to assess the relative increase/decrease between two cities. Relatively small counts in Brisbane could be the reason why we obtained extremely large or small point estimates for the post-SARS RR of CRD hospitalizations and the post-pandemic RR of stroke mortality in Brisbane; hence, these RRs need to be interpreted with caution. Unfortunately, good quality mortality, hospitalization, influenza surveillance and vaccination data are available in few subtropical countries/regions. Nevertheless, this study is the first to investigate the effectiveness of influenza and pneumococcal vaccination at the population level in warm climates, to our best knowledge.

There are several limitations in this study. First, ecological fallacy is unavoidable given the ecological study design. Individual vaccination status of those who have died or been hospitalized is unknown and the outcome variables are not specific to influenza. Nevertheless, we have used a previously validated modeling approach to estimate disease burden associated with influenza. Second, we assume that circulating influenza strains and pre-exisitng immunity at the population level are similar between Hong Kong and Brisbane. Therefore, a relative decrease (or less of an increase) in influenza-associated disease burden could reflect the effectiveness of influenza vaccination in terms of reducing adverse outcomes after influenza infections. This assumption may not hold, but there is also no strong evidence against it. Third, only two to five years of data were included in each study period, because influenza virology and hospitalization data prior to 2000 were not available in Hong Kong or Brisbane. Our model obtained some unstable points estimates, especially in the pre-SARS period, which could be due to the short time series and low counts. Last but not least, although we have carefully adjusted for seasonal trends, temperatures, and humidity in our models, there are many confounding factors that remain unadjusted for in this study, such as the prevalence of underlying condition, and difference in health-seeking behaviors between two older populations.

In conclusion, we found some but limited evidence that markedly increased rates of influenza and pneumococcal vaccination among the Hong Kong older people did lead to a reduction in their influenza disease burden. However, furture cohort studies with individual data are warranted to provide stronger evidence to support the promotion of influenza vaccination among the older population.

## Additional file

Additional file 1: **Appendix 1.** Additional information on data sources and statistical analysis. **Appendix 2.** Study periods defined for Hong Kong and Brisbane. **Appendix 3.** Time series plots of weekly numbers of cause-specific mortality data of Hong Kong (black line) and Brisbane (gray line). **Appendix 4.** Time series plots of weekly numbers of hospital admissions of Hong Kong (black line) and Brisbane (gray line). Hospitalization data of Brisbane are not available in the post-pandemic period. **Appendix 5.** Time series plots of weekly mean temperature and relative humidity Hong Kong (black line) and Brisbane (gray line). **Appendix 6.** Annual excess rates of mortality and hospitalizations associated with influenza per 1000,000 population in Hong Kong and Brisbane, 2001–2012. (DOCX 484 kb)

## Abbreviations

AER: Annual excess rate
CI: Confidence interval
COPD: Chronic obstructive pulmonary disease
CRD: Cardiorespiratory diseases
ER: Excess rates
IHD: Ischemic heart diseases
P&I: Pneumonia and influenza
RR: Rate ratio
SARS: Severe Acute Respiratory Syndrome
