# Supplementary material for: Comparison of influenza disease burden in older populations of Hong Kong and Brisbane: the impact of influenza and pneumococcal vaccination
Source: BMC Infect Dis. 2019 Feb 14;19:162. doi: 10.1186/s12879-019-3735-7 (PMC6376732; doi:10.1186/s12879-019-3735-7)
Supplement: Supplementary file 1 — Appendix 1. Additional information on data sources and statistical analysis. Appendix 2. Study periods defined for Hong Kong and Brisbane. Appendix 3. Time series plots of weekly numbers of cause-specific mortality data of Hong Kong (black line) and Brisbane (gray line). Appendix 4. Time series plots of weekly numbers of hospital admissions of Hong Kong (black line) and Brisbane (gray line). Hospitalization data of Brisbane are not available in the post-pandemic period. Appendix 5. Time series plots of weekly mean temperature and relative humidity Hong Kong (black line) and Brisbane (gray line). Appendix 6. Annual excess rates of mortality and hospitalizations associated with influenza per 1000,000 population in Hong Kong and Brisbane, 2001–2012. (DOCX 484 kb) [file 12879_2019_3735_MOESM1_ESM.docx]

**Appendix 1. Additional information on data sources and statistical analysis.**

We obtained weekly figures on the number of specimens that had tested positive for influenza A and B as well as on the total number of specimens collected in Hong Kong from the microbiology laboratory of Queen Mary Hospital (QMH). These data have been shown representative of the entire territory in the previous study[1]. The virology data for Brisbane during the period from 30 April 2001 to 1 January 2010 were obtained from Queensland Health Australia, but only weekly positive numbers for influenza A and B were available. After that period, the virology data for the city of Brisbane were not available, so we obtained virology data for the state of Queensland for the period from 2 January 2010 to 16 December 2012 from the Department of Health of the Australian Government, to approximate the activity of the virus in Brisbane. These two datasets show a high correlation during the overlapping period of year 2009 (Pearson correlation coefficient = 0.91, p<0.001).

To calculate the influenza associated mortality and morbidity burden, death registry data for Hong Kong and Brisbane were obtained from the Census and Statistics Department of Hong Kong and the Australian Institute of Health and Welfare, respectively. These data were aggregated into weekly numbers of deaths from the following underlying causes: all-causes (ICD10 A00-T99), cardio-respiratory disease (CRD, ICD10 I00-J99), pneumonia and influenza (P&I, ICD10 J09-J18), chronic obstructive pulmonary disease (COPD, ICD10 J40-J47), stroke (ICD9 430-438; ICD10 I60-I69), and ischemic heart diseases (IHD, ICD10 I20-I25). Hospital admission data were obtained from the Hospital Authority of Hong Kong and Queensland Health. Weekly numbers of hospital admissions were aggregated by the discharge diagnosis of CRD (ICD9 390-459), acute respiratory disease (ARD, 460-519), P&I (ICD9 480-487), COPD (ICD9 490-496), stroke (ICD9 001-999), and IHD (ICD9 410-414). Weekly numbers of deaths and admissions from injuries and poisoning (ICD9 800-999 and ICD10 S00-T98) in Hong Kong were also collected as a control disease category, since accidents were theoretically not related to influenza infections.

Meteorological data, including daily mean temperatures and relative humidity, from 2001 to 2013 were obtained from the Hong Kong Observatory and the Australian Bureau of Meteorology.

A typical form for the time series segmented regression models is:

$$\log\left( Y_{t} \right)=a_{1}+offset\left( \log\left( pop_{t} \right) \right)+S\left( {time}_{t}, {df}_{1} \right)+S\left( {temp}_{t}, {df}_{2} \right)+S\left( {hum}_{t}, {df}_{3} \right)+SARS+PostSARS+Pandemic+PostPandemic+flu_{t}+flu_{t}\times SARS+flu_{t}\times PostSARS+flu_{t}\times Pandemic+flu_{t}\times PostPandemic$$

Here, *Yt* denotes weekly numbers of cause-specific mortality or hospitalization in Hong Kong or Brisbane, $flu_{t}$denotes the proxy variable for influenza activity (weekly percentage of positive specimens in the annual total number of positive samples). Four dummy variables for the SARS, post-SARS, pandemic, and post-pandemic periods, and their interaction terms with influenza proxies were added into the model to estimate the effects of influenza for each study period. The natural spline of seasonal and long-term trends $S\left( {time}_{t}, {df}_{1} \right)$, temperature $S\left( {temp}_{t}, {df}_{2} \right)$, and relative humidity $S\left( {hum}_{t}, {df}_{3} \right)$ were added as confounders. An offset variable of $offset\left( \log\left( pop_{t} \right) \right)$was added to adjust for the differences in population size. For the models that were fitted to hospitalization data, the annual total number of hospital beds in public hospitals and the weekly numbers of total admissions were also included as confounders.

Degrees of freedom ranged from 2 to 24 for time and from 3 to 4 for temperature and relative humidity in the Poisson models fitted to different outcomes.

The best-fitting models were chosen by minimal generalized cross-validation (GCV), according to our previous study [2]. Baseline rates of cause-specific mortality and hospitalizations associated with influenza were calculated for different periods by setting the virus proxy to zero and the corresponding period dummy to one (other dummies were simultaneously set to zero). Excess numbers were derived by subtracting baseline rates from the observed data, and excess rates (ER) were further calculated by dividing the excess numbers by the size of the age-specific population.

The 95% confidence interval (CI) of ER was calculated by bootstrapping 1,000 times. Because the periods were of different lengths, we calculated annual rates of excess mortality (or hospitalizations) (AER) to facilitate comparisons between different periods. For each disease category, the rate ratio (RR) of post-SARS (or post-pandemic) versus pre-SARS was derived by dividing annual excess rates during the post-SARS (or post-pandemic) period with those of the pre-SARS period (as reference):

*RR = AER (post-SARS) / AER (pre-SARS)*

Since the pre-SARS period was treated as the reference period in this study, hereafter the post-SARS RR refers to the risk ratio of mortality or hospitalization in the post-SARS period relative to those in the pre-SARS period. Similarly, the post-pandemic RR refers to the risk ratio of mortality or hospitalization in the post-pandemic period relative to those in the pre-SARS period. The 95% CI and *p*-value of RR were derived from a normal approximation of their logarithmic transformations [3].

We also conducted several sensitivity analyses to test the robustness of our model estimates. First, we narrowed down the study periods to influenza peak seasons only. The influenza season was defined as being from January to July in Hong Kong, and from May to November in Brisbane. Second, we conducted a sensitivity analysis by excluding the data for the mismatched years (the years 2003, 2004, and 2008 in this study).

**References**

1. Yang L, Wong CM, Chan KP, Chau PY, Ou CQ, Chan KH, Peiris JS: **Seasonal effects of influenza on mortality in a subtropical city**. *BMC infectious diseases* 2009, **9**:133.

2. Wang XL, Yang L, Chan KP, Chiu SS, Chan KH, Peiris JS, Wong CM: **Model selection in time series studies of influenza-associated mortality**. *PloS one* 2012, **7**(6):e39423.

3. Rothman KJ, Greenland S: **Modern Epidemiology**: Lippincott-Raven 1998.

**Appendix 2. Study periods defined for Hong Kong and Brisbane.**

| City | Period | Start Date | End Date | No of weeks |
| --- | --- | --- | --- | --- |
| Hong Kong | Pre-SARS | 31/12/2000 | 29/12/2002 | 104 |
|  | SARS | 30/12/2002 | 02/08/2003 | 31 |
|  | Post-SARS | 03/08/2003 | 25/04/2009 | 299 |
|  | Pandemic | 26/04/2009 | 31/07/2010 | 66 |
|  | Post-pandemic | 01/08/2010 | 29/12/2012 | 126 |
| Brisbane | Pre-SARS | 30/04/2001 | 29/12/2002 | 87 |
|  | SARS | 30/12/2002 | 03/08/2003 | 31 |
|  | Post-SARS | 04/08/2003 | 26/04/2009 | 299 |
|  | Pandemic | 27/04/2009 | 01/08/2010 | 66 |
|  | Post-pandemic | 02/08/2010 | 16/12/2012 | 124 |

**Appendix 3. Time series plots of weekly numbers of cause-specific mortality data of Hong Kong (black line) and Brisbane (gray line).**


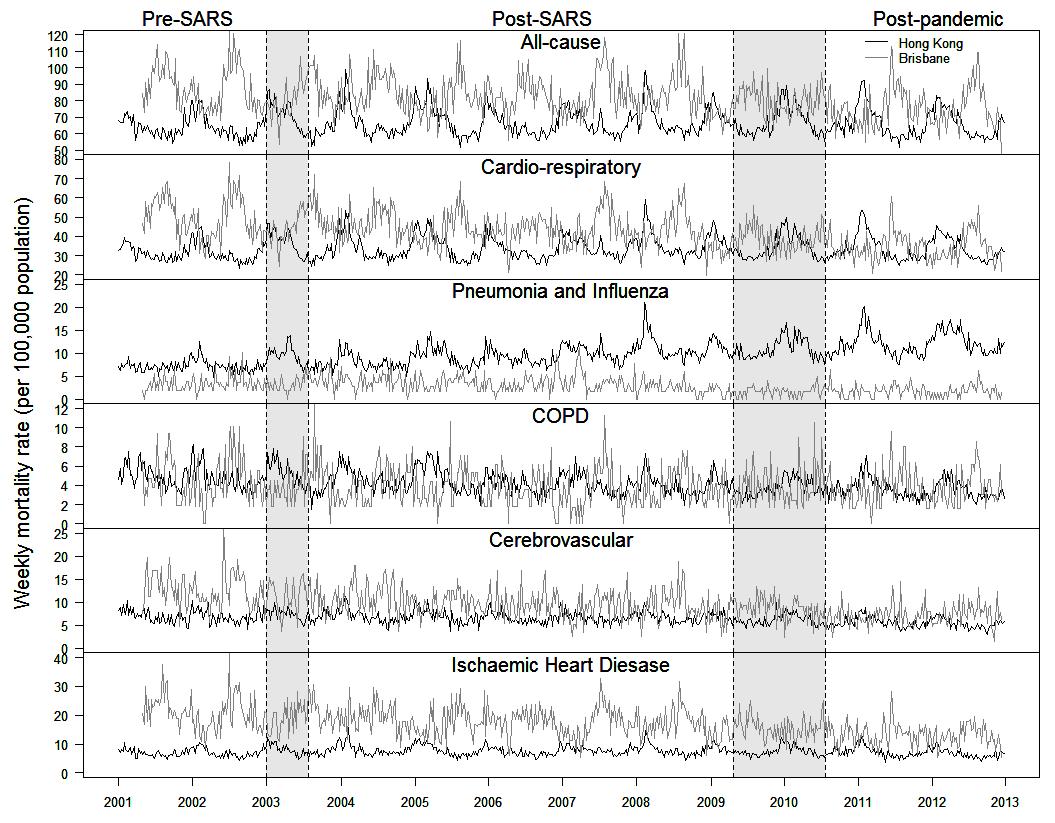


**Appendix 4. Time series plots of weekly numbers of hospital admissions of Hong Kong (black line) and Brisbane (gray line). Hospitalization data of Brisbane are not available in the post-pandemic period.**


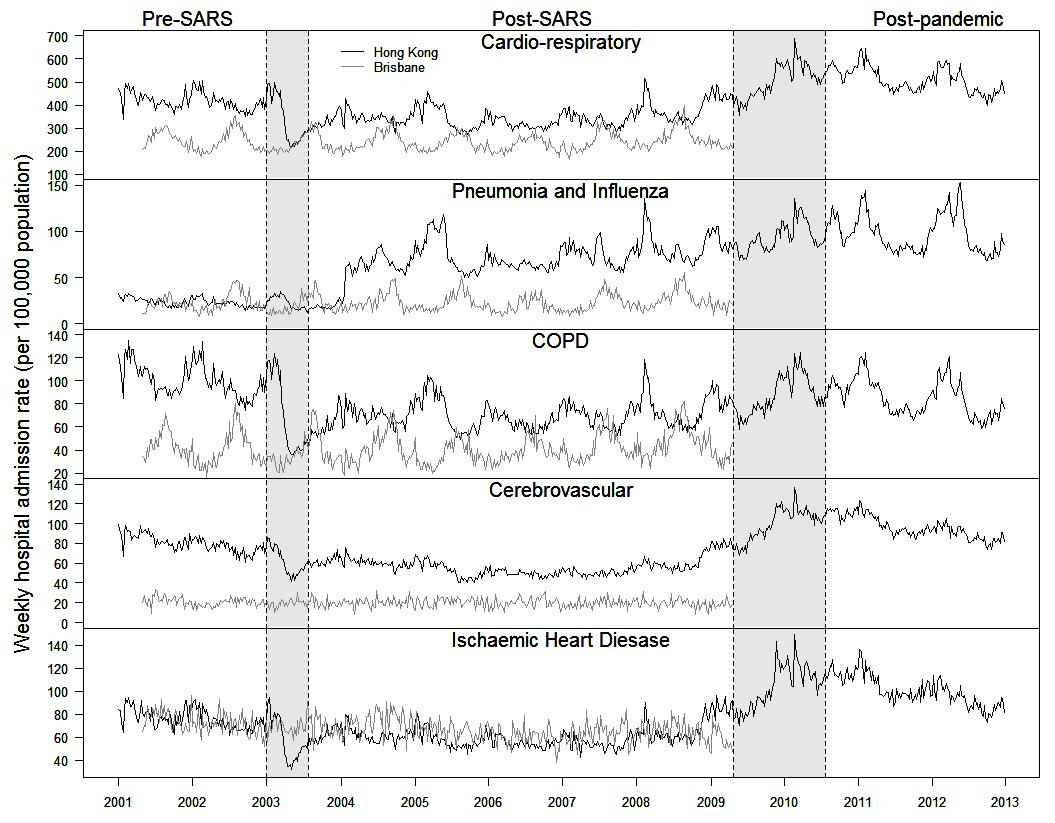


**Appendix 5. Time series plots of weekly mean temperature and relative humidity Hong Kong (black line) and Brisbane (gray line).**


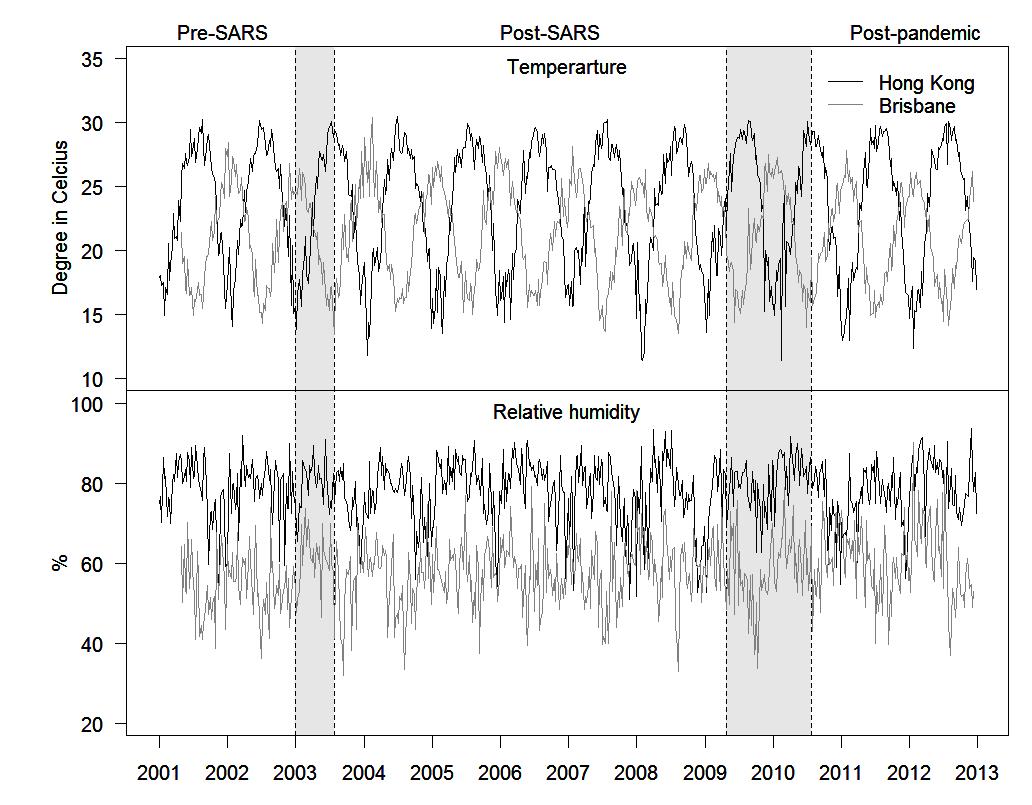


**Appendix 6. Annual excess rates of mortality and hospitalizations associated with influenza per 1,000,000 population in Hong Kong and Brisbane, 2001-2012.**

| Mortality | Pre-SARS | |  | Post-SARS | |  | Post-pandemic | | *p* |  |
| --- | --- | --- | --- | --- | --- | --- | --- | --- | --- | --- |
|  | rate | (95% CI) | *p* | rate | (95% CI) | *p* | rate | (95% CI) |  |  |
| Hong Kong mortality | | | | | | | | | | |
| All-causes | 813 | (282, 1,411) | 0.004 | 988 | (756, 1,210) | <0.001 | 1147 | (851, 1,444) | <0.001 |  |
| CRD | 725 | (356, 1,131) | <0.001 | 650 | (485, 806) | <0.001 | 840 | (632, 1,061) | <0.001 |  |
| P&I | 134 | (-24, 290) | 0.086 | 336 | (269, 402) | <0.001 | 327 | (225, 436) | <0.001 |  |
| COPD | 112 | (-2, 237) | 0.054 | 117 | (72, 157) | <0.001 | 120 | (70, 166) | <0.001 |  |
| Stroke | 72 | (-51, 205) | 0.268 | 53 | (1, 102) | 0.048 | 90 | (28, 145) | <0.001 |  |
| IHD | 210 | (67, 356) | 0.006 | 94 | (26, 155) | 0.004 | 155 | (80, 228) | <0.001 |  |
| Injuries | 25 | (-31, 90) | 0.403 | 30 | (-40, 124) | 0.378 | 15 | (-55, 119) | 0.718 |  |
| Brisbane mortality | | | | | | | | | | |
| All-cause | 679 | (107, 1,283) | 0.014 | 590 | (271, 894) | <0.001 | 1621 | (941, 2,354) | <0.001 |  |
| CRD | 558 | (114, 1,015) | 0.004 | 440 | (198, 655) | <0.001 | 545 | (80, 1,039) | 0.028 |  |
| P&I | -32 | (-140, 73) | 0.570 | 14 | (-42, 72) | 0.686 | 101 | (-4, 197) | 0.062 |  |
| COPD | 96 | (-21, 220) | 0.110 | 99 | (42, 157) | <0.001 | 168 | (29, 306) | 0.016 |  |
| Stroke | 173 | (-30, 366) | 0.094 | 56 | (-45, 147) | 0.248 | 50 | (-161, 245) | 0.594 |  |
| IHD | 213 | (-48, 481) | 0.112 | 231 | (92, 357) | <0.001 | -71 | (-353, 214) | 0.662 |  |
| Hong Kong hospitalizations | | | | | | | | | | |
| CRD | 4002 | (2,096, 6,138) | <0.001 | 3450 | (2,761, 4,145) | <0.001 | 3075 | (1,894, 4,341) | <0.001 |  |
| P&I | 955 | (425, 1,511) | <0.001 | 2668 | (2,301, 3,046) | <0.001 | 3782 | (3,202, 4,411) | <0.001 |  |
| COPD | 1299 | (424, 2,220) | <0.001 | 1356 | (1,052, 1,653) | <0.001 | 1446 | (995, 1,962) | <0.001 |  |
| Stroke | 769 | (229, 1,326) | 0.004 | 118 | (-53, 289) | 0.188 | -131 | (-437, 202) | 0.106 |  |
| IHD | 267 | (-320, 833) | 0.370 | -36 | (-212, 177) | 0.760 | -72 | (-417, 247) | 0.720 |  |
| Injuries | -429 | (-912, 69) | 0.091 | -233 | (-813, 378) | 0.447 | -119 | (-1082, 917) | 0.816 |  |
| Brisbane hospitalizations | | | | | | | | | | |
| CRD | 34 | (-816, 921) | 0.980 | 923 | (486, 1,337) | <0.001 | NA |  |  |  |
| P&I | 136 | (-265, 532) | 0.526 | 475 | (281, 660) | <0.001 | NA |  |  |  |
| COPD | 436 | (-96, 894) | 0.126 | 579 | (340, 806) | <0.001 | NA |  |  |  |
| Stroke | 5 | (-243, 263) | 0.996 | -4 | (-151, 139) | 0.976 | NA |  |  |  |
| IHD | -606 | (-1,062, -128) | 0.010 | -301 | (-562, -57) | 0.006 | NA |  |  |  |

Note. NA, not available. Hospitalization data in Brisbane after 2009 are not available to us.
